# No Differences in Emotion Recognition Strategies in Children with Autism Spectrum Disorder: Evidence from Hybrid Faces

**DOI:** 10.1155/2014/345878

**Published:** 2014-01-05

**Authors:** Kris Evers, Inneke Kerkhof, Jean Steyaert, Ilse Noens, Johan Wagemans

**Affiliations:** ^1^Laboratory of Experimental Psychology, KU Leuven, Tiensestraat 102 (Box 3711), 3000 Leuven, Belgium; ^2^Department of Child Psychiatry, UPC-KU Leuven, 3000 Leuven, Belgium; ^3^Leuven Autism Research (LAuRes), KU Leuven, 3000 Leuven, Belgium; ^4^Quantitative Psychology and Individual Differences, KU Leuven, 3000 Leuven, Belgium; ^5^Department of Clinical Genetics, University Hospital Maastricht, 6200 Maastricht, The Netherlands; ^6^Parenting and Special Education Research Unit, KU Leuven, 3000 Leuven, Belgium; ^7^Psychiatric and Neurodevelopmental Genetics Unit, Massachusetts General Hospital, MA, Boston, USA

## Abstract

Emotion recognition problems are frequently reported in individuals with an autism spectrum disorder (ASD). However, this research area is characterized by inconsistent findings, with atypical emotion processing strategies possibly contributing to existing contradictions. In addition, an attenuated saliency of the eyes region is often demonstrated in ASD during face identity processing. We wanted to compare reliance on mouth versus eyes information in children with and without ASD, using hybrid facial expressions. A group of six-to-eight-year-old boys with ASD and an age- and intelligence-matched typically developing (TD) group without intellectual disability performed an emotion labelling task with hybrid facial expressions. Five static expressions were used: one neutral expression and four emotional expressions, namely, anger, fear, happiness, and sadness. Hybrid faces were created, consisting of an emotional face half (upper or lower face region) with the other face half showing a neutral expression. Results showed no emotion recognition problem in ASD. Moreover, we provided evidence for the existence of top- and bottom-emotions in children: correct identification of expressions mainly depends on information in the eyes (so-called top-emotions: happiness) or in the mouth region (so-called bottom-emotions: sadness, anger, and fear). No stronger reliance on mouth information was found in children with ASD.

## 1. Introduction

Facial expressions inform us about the feelings and state of mind of others and enable us to adjust our behavior and to react appropriately. Therefore, the ability to interpret facial expressions accurately and to derive socially relevant information from them is considered a fundamental requirement for typical reciprocal social interactions and communication [1].

Autism spectrum disorders (ASD) are pervasive developmental disorders characterized by quantitative and qualitative deficits in reciprocal social interactions and communication and by the presence of restricted and repetitive behavior patterns, interests, and activities [2]. Difficulties in recognizing, identifying, and understanding the meaning of emotions are often considered as one of the trademarks of their social problems. Different procedures have been used to examine emotion processing abilities in children and adults with ASD, with or without intellectual disability: sorting, (cross-modal) matching, and labeling tasks (for a literature review and a meta-analysis on this topic, see [3, 4], resp.). Each of these procedures has revealed problems with affect processing in individuals with ASD (e.g., [5–8]). Other studies, however, failed to find atypical emotion recognition skills in individuals with ASD (e.g., [9–13]). Inconsistencies (for an overview, see [3]) may be due to differences in sample and participants' characteristics, task demands [14], and stimuli. Performances of individuals with ASD seem to be especially impaired for negative, more subtle, or more complex emotions (e.g., [7, 15, 16]) or expressions embedded in a social context [17, 18]. Besides, it is important to note that an intact performance does not exclude the use of atypical—more cognition- or language-mediated—or more feature-based emotion processing strategies leading to impairments in some emotion recognition tasks, but camouflaging these deficits in other tasks (and therefore often referred to as compensation mechanisms; [3, 11, 19]).

Interestingly, face identity research has provided evidence for atypical face processing strategies in individuals with ASD (for a review, see [20]). People with ASD show an attenuated reliance on the eyes region: they are less attentive towards the upper face part in comparison to neurotypicals, but instead the lower face seems to be more salient. This was found using face identification tasks with familiar [21] and unfamiliar faces (e.g., [22, 23]) and with eye-movement studies using neutral faces or faces embedded in a social context (e.g., [24, 25]). This atypical attention pattern with an increased “mouth bias” in ASD was sometimes interpreted as a specific expertise for mouth regions [26], or as a (language-and-cognition mediated) compensation strategy [27], or even as an active aversion for the quickly and unpredictably moving eyes [28]. However, not all studies found differences in gaze patterns between children with and without ASD (e.g., [29, 30]). Here also, inconsistencies are supposed to be caused by differences in samples, tasks, and stimuli [3].

The above-mentioned studies concern facial identity processing. It is sometimes suggested that face identity recognition (which is based on invariant face properties) and face expression recognition (which is based on changeable aspects of a face) can be dissociated at a neural and theoretical level [31, 32]. According to this view, findings about face identity processing will not necessarily generalize to the domain of emotion perception (see also findings on patients with amygdalectomy [33] or acquired prosopagnosia [34]). The aim of the present study is to examine whether the atypical saliency of upper versus lower face area also holds for emotion perception in children with ASD.

Attendance to a narrow range of specific features in the face (e.g., the mouth region) when judging facial affect could partly explain the difficulties that children with ASD experience in reading emotional expressions. Some evidence for increased reliance on mouth information and less sensitivity for the eyes was already found ([16, 27, 35, 36], but also see, e.g., [37]). A disturbed attention pattern might affect emotion recognition performance most strongly in emotions where information of the eyes is more crucial. Research in typical individuals provided evidence for a distinction between top-emotions (most salient information in the eyes region) and bottom-emotions (most salient information in the mouth region). Behavioral research in adults demonstrated that the recognition of sadness, anger, and fear mostly relies on information in the eyes region, and the recognition of happiness and disgust relies on information in the mouth region ([38], Experiment 1). More recently, eye-movement studies also demonstrated that the most characteristic face regions (top or bottom) depend on nature of the emotional expression [39–41].

**Figure 1:**
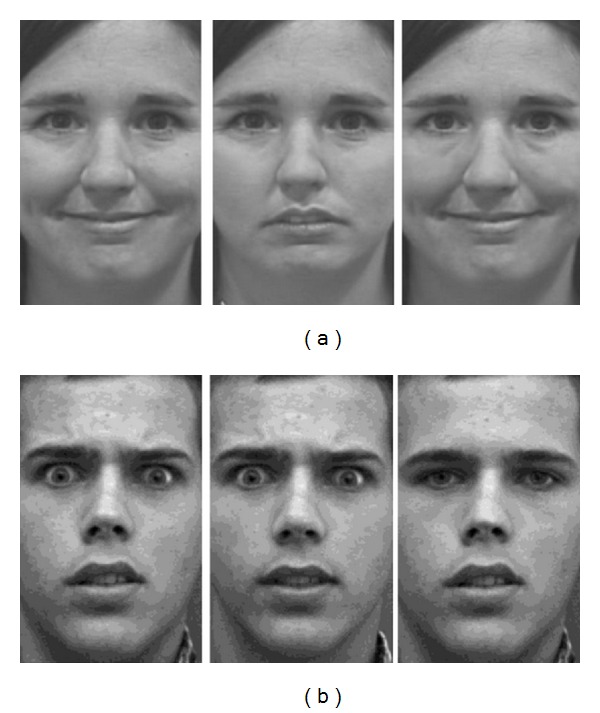
Representative sample stimulus of bottom-emotion happiness (a) and top-emotion fear (b), from left to right: original expression, hybrid expression with neutral mouth, and hybrid expression with neutral eyes (the pictures used in our paper are part of the California Facial Expression database, which is freely available online for research purpose. For more information regarding this database, we refer you to their website: http://cseweb.ucsd.edu/users/gary/CAFE/readme.txt).

Using an emotion labeling task with hybrid stimuli, we wanted to compare the reliance on eyes versus mouth information in children with and without ASD when recognizing emotional expressions. Hence the original expressions of anger, fear, happiness, and sadness and neutral expressions were shown together with their hybrid counterparts. The hybrid facial expressions used here are computer-morphed emotional expressions with the upper (or lower) face half being replaced by a neutral expression, such that emotional information is only available in the mouth (or eyes, resp.) region (see Figure 1 for an example). The generation of hybrid facial stimuli is a rather novel technique with some significant benefits in comparison to eye-movement studies. Whereas both methods aim at evaluating automatic and implicit emotion recognition strategies, the most salient advantage of hybrid stimuli is a practical one. An expensive eye-tracking setup is obviously not required, making the experimental design easier to implement, and also significantly reduces the burden on participants. The data obtained within the context of an eye-movement experiment are clearly richer, yet much more difficult and less clear-cut to process. In addition to these more pragmatic advantages of using hybrid stimuli, an important methodological difference is the active manipulation of available information in hybrid expressions. Showing full facial expressions allows participants to (freely) scan the whole picture. However, this is impossible when using hybrid expressions, simply because no all emotional information is available. Clearly, both paradigms offer a slightly different perspective on the evaluation of saliency of upper versus lower facial areas. Eye-movement studies indeed provide a stronger indication of spontaneous viewing strategies, but the drawback is that participants can adapt their viewing style, with both strategies being impossible to disentangle. Hybrid facial expressions, on the other hand, offer the opportunity to evaluate the reliance on a specific face half when recognizing emotions, by comparing performances on full expressions and their hybrid counterparts. Studies using face parts, such as, for instance, the part-whole paradigm or composite face illusions, also actively intervene in the available information. However, the lack of naturalness is a major disadvantage of using face parts. The processing of these nonfacial and artificial stimuli might be unnatural anyway, not telling us much about (full) face perception.

This study had two goals. We firstly wanted to evaluate whether children with ASD would experience difficulties when labeling four basic emotional expressions and a neutral expression. Secondly, we aimed at comparing eyes versus mouth reliance between children with and without ASD. If typically developing children mostly relied on information in the eyes region, they were expected to experience more difficulties recognizing a hybrid expression with neutral eyes, and especially for those emotions with more characteristic features in the eyes region (top-emotions), such as fear, anger, and sadness. An attenuated bias for the eyes region in the ASD group, or in increased reliance on the mouth area, would result in the reverse pattern. The ASD sample would then be mostly impaired when recognizing hybrid expressions with neutral mouths, and especially for bottom-emotions which have more salient features in the mouth area, such as happiness.

## 2. Methods

### 2.1. Participants

Two groups of six-to-eight-year-old boys without an intellectual disability (IQ ≥ 70) participated in this study: an experimental group and a matched comparison group. The experimental group consisted of 22 boys with ASD, recruited through the child psychiatry unit at the university hospital (*n* = 16) and through a special needs school for children with ASD (*n* = 6). They all received a formal clinical diagnosis of ASD based on a comprehensive assessment by a multidisciplinary team (first subgroup) or by a medical specialist (second subgroup), and according to DSM-IV-TR criteria [2]. Clinical diagnoses were confirmed within the research protocol using the Autism Diagnostic Interview-Revised (ADI-R; [42]) and by file review by a child psychiatrist experienced in ASD. Whereas all diagnoses were confirmed based on file review, all but two participants scored above cut-off on the reciprocal social interactions and the language/communication ADI-R domains. Four additional patients did not meet the restrictive and repetitive behaviors ADI-R domain. In the ASD group, full-scale IQ was measured within the recent clinical diagnostic protocol using the Wechsler Scales of Intelligence (WISC-III-R; [43]) or the Snijders-Oomen Non-Verbal Intelligence Test (SON-R; [44]).

The comparison group comprised 22 typically developing boys (TD group), representative for the general population, and recruited through two mainstream schools. Within the experimental protocol, full-scale IQ was estimated based on an abbreviated version of the WISC-III-R [45], comprising four subtests (vocabulary, similarities, picture arrangement, and block design). The ASD and TD groups were groupwise matched based on age and full-scale IQ (for detailed participant characteristics and group comparisons, see Table 1).

### 2.2. Materials

Black-and-white photographs of twelve adult faces (seven males and five females) were selected from the California Facial Expressions database [46]. These stimuli were posed according to the Facial Action Coding System (FACS; [47]) and were normalized for the location of eyes and mouth. Hybrid facial expressions were created with Adobe Photoshop. In a hybrid expression, the upper (or lower) face part is replaced by a neutral upper (or lower) face of the same actor. For every emotion-identity combination, two types of hybrid expressions were made: one in which the upper part was replaced by a neutral expression (“hybrid with neutral eyes”) and one in which the lower part was replaced by a neutral expression (“hybrid with neutral mouth”). The clone sample tool was used as smoothing tool to mask the lines between both face halves (see Figure 1 for an example).

Five different facial expressions were used: one neutral expression and four emotional faces, expressing happiness, sadness, anger, and fear. Based upon a pilot study (with six TD children and eight TD adults), the three (out of four) best recognizable emotions were selected for every actor, in such a way that, in total, all emotional expressions were presented the same number of times (i.e., all four emotions were presented nine times).

### 2.3. Procedure and Design

Approval by the Medical Ethical Committee of the University Hospital was obtained and the participants' parents gave their written informed consent before start. All participants were tested individually in a quiet room. Stimuli were presented on a portable computer with a 38 cm screen, at a viewing distance of approximately 76 cm. Stimuli were presented within the E-prime environment. The experimental procedure followed a step-by-step protocol, including a phase wherein participants were made familiar with the different response alternatives and their understanding of the (emotion) labels was checked. This was done by asking for an example situation for the different emotions. If participants could not provide a correct example situation, the experimenter presented a standard example, as outlined in the protocol. Children were instructed to label the facial expressions. All participants understood the task and the emotion labels. Trials consisted of a face stimulus (presented at the left side) and the response alternatives (presented at the right side). Participants responded verbally or by pointing to the label at the screen. Experimenters always entered the participant's response. In between two trials a blank screen with fixation cross was presented for one second. The face stimuli were presented with an unlimited presentation time.

In total, this experiment consisted of 120 trials, so that each expression was presented 10 times per identity: 12 neutral facial expressions (1 per identity) + 36 original emotional expressions (3 emotions × 12 identities) + 72 hybrid emotional expressions (3 emotions × 12 identities × 2 hybrid types, namely, neutral mouth and neutral eyes). Trial order was randomized.

### 2.4. Data and Data Analysis

All analyses were performed with SAS. Split-plot ANOVAs were conducted on accuracy scores. Accuracy data for the original expressions were analyzed separately, with group (ASD-TD group) as between-subjects factor and expression (neutral-happiness-sadness-anger-fear) as within-subjects factor. In a second analysis, all versions, the hybrid and the original expressions, were analyzed, with the neutral expressions being omitted from these analyses because they had no hybrid counterpart. Again, a split-plot ANOVA with group (ASD-TD group) as between-subjects factor and emotional expression (happiness-sadness-anger-fear) and hybrid type (hybrid neutral mouth-hybrid neutral eyes-original emotional expression) as within-subjects factors was conducted for accuracy data. A significance level of *P* < .05 (two-sided) was adopted, and effects with a *P* value between .05 and .10 were indicated as marginally significant.

All analyses performed on the whole ASD group (*n* = 22) yielded similar results as analyses with the subgroup of ASD participants who scored above the cut-off on the social and communication domain of the ADI-R (*n* = 20), or on those participants who scored above cut-off on all ADI-R domains (*n* = 16). Therefore, we will report the results of the analyses on the whole group.

## 3. Results

### 3.1. Recognition of the Original Expressions and Relationship with Age, Full-Scale IQ, or ASD Severity

**Figure 2:**
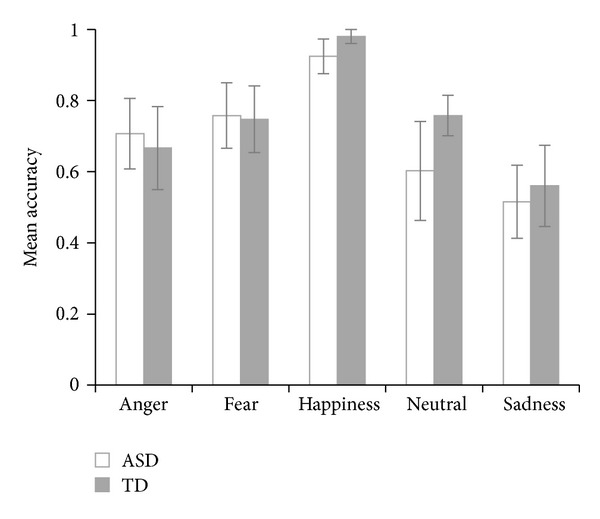
Mean performances for both participant groups on the different original expressions (error bars represent 95% confidence intervals of the means).

Results (see Table 2) indicated a main effect of expression (*F*(4,168) = 27.28, *P* < .0001, see Figure 2). Post hoc Tukey-Kramer tests (*α* = .05) revealed that sadness (*M* = .54) was overall recognized less accurately than all other emotions, and happiness (*M* = .95) was overall recognized more accurately than all other emotions. We did not find evidence for a main effect of participant group (*F*(1,42) = 1.14, *P* = .29) and the participant group-by-expression-interaction was not significant either (*F*(4,168) = 1.69, *P* = .15).

No evidence for a correlation between emotion recognition and age, intelligence, or ASD traits (measured with the ADI-R) was found (all correlations with *P* values > .10).

### 3.2. Which Face Region Is Used to Recognize Emotional Expressions: Mouth or Eyes?

Again, sadness was recognized less accurately (across group and hybrid type) than the other expressions, and happiness was the best recognized emotion (*F*(3,126) = 34.35, *P* < .0001; post-hoc Tukey-Kramer tests). Evidence for a main effect of hybrid type was also found (*F*(2,84) = 78.14, *P* < .0001), such that original stimuli were recognized the most accurate, and hybrid expressions with neutral eyes were recognized the least accurate. Interestingly, the interaction between emotion and hybrid type was highly significant (*F*(6,252) = 57.28, *P* < .0001), indicating that the most characteristic region (eyes or mouth) is emotion-dependent. Post hoc Bonferroni adjusted tests supported the distinction between top-emotions (anger, fear, and sadness) and bottom-emotions (happiness). Moreover, these tests indicated that adding the other, less characteristic face half did not significantly improve the performance (i.e., no statistically significant difference between the recognition of original expressions and the most salient hybrid ones).

**Figure 3:**
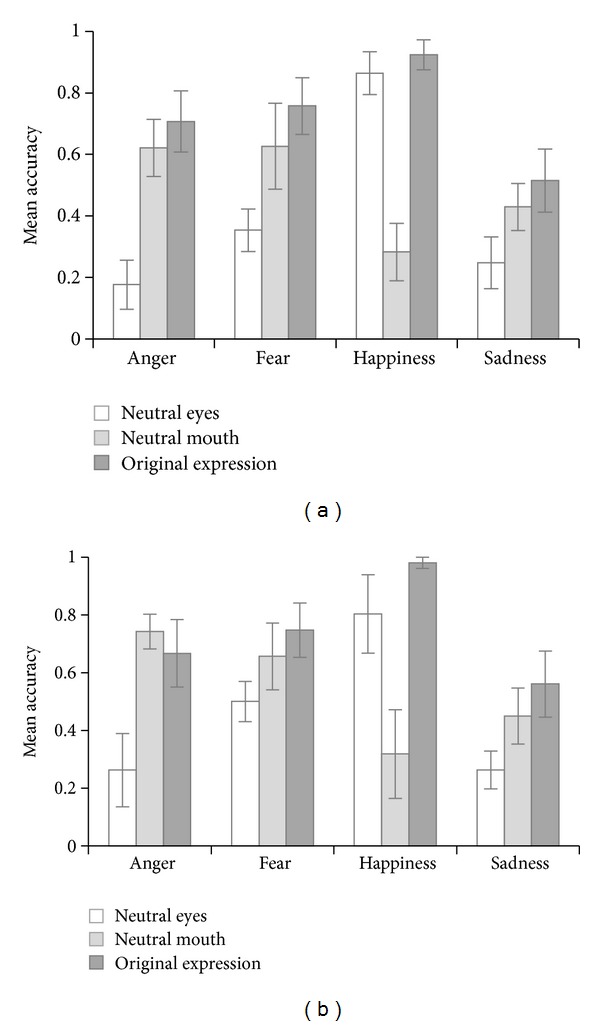
The effect of stimulus presentation (hybrid expression with neutral eyes, hybrid expression with neutral mouth, and original expression) on emotion recognition performance was similar in both the ASD group (a) and in the TD group (b). Happiness is a bottom-emotion; sadness, anger, and fear are top-emotions. Error bars represent 95% confidence intervals of the means.

The main effect of participant group was not significant (*F*(1,42) = 2.31, *P* = .14), nor were the interactions between participant group and emotion, or between participant group and hybrid type (all *F* < 1). Contrary to our expectations, the 3-way interaction among participant group, emotion, and hybrid type was not significant (*F*(6,252) = 1.59, *P* = .15, depicted in Figure 3 and Table 3). This suggests that both children with and without ASD show a similar distinction between expressions for which the mouth region contains the most salient information (bottom-emotions) and expressions for which the eyes region is the most informative (top-emotions).

## 4. Discussion

The goal of this research was to investigate (1) whether children with ASD were impaired in recognizing facial expressions of four basic emotions and a neutral one and (2) whether children with ASD tend to rely more on features in the lower part of the face (mouth region) when recognizing emotions. We evaluated this with a novel stimulus generation technique, namely, hybrid expressions, hereby actively manipulating the amount of emotional information available in the face stimulus. A computer-morphed facial expression was generated, with the upper (or lower) face half being replaced by a neutral expression, such that emotional information is only present in the other face half (lower or upper region, resp.).

No impairment in the recognition of angry, happy, sad, and fearful and neutral original expressions was found in children with ASD, compared to an age- and IQ-matched group of TD children. Both participant groups performed well above chance on these original expressions, which indicated that our stimulus selection was successful. Indeed, it was necessary for this study to use expressions with a high recognition rate, so that correct identification was still feasible based on a reduced amount of information (when recognizing the hybrid emotions). Although some studies found children with ASD to be outperformed by TD children in basic emotion recognition tasks, nowadays it has been generally accepted that children with ASD (without intellectual disability) mostly succeed in structured emotion processing tasks, using basic and easily recognizable stimuli [3]. In line with this, a challenge for future applied research is to develop experimental procedures that can tap into the more profound understanding of the meaning of emotions. Besides, one should evaluate whether existing emotion training programs for children with ASD teach them more than mere labelling the basic affects correctly.

Interestingly, we found an interaction between emotion and hybrid type, providing evidence for the existence of top- and bottom-emotions in children. Mouth information holds crucial information to correctly identify the emotion happiness. For the emotions anger, fear, and sadness, the most salient information is located in the region of the eyes. Hereby we nicely replicate and extend the previously demonstrated distinction between top- and bottom-emotions to a group of children with and without a developmental disorder (studies in adults: [38–40]). At the same time, this effect demonstrates that the experiment had sufficient statistical power to pick up subtle performance differences. Moreover, it proves that this method—using hybrid stimuli—is valuable for a coarse evaluation of attentional bias, without the need for eye-movement registration.

In general, original expressions were recognized more accurately than hybrid faces with neutral eyes and hybrid faces with neutral mouth. However, this effect can be mainly attributed to the apparent overrepresentation of top-emotions in the stimulus selection. In addition, providing the most salient or relevant emotional information (mouth or eyes region, for bottom- and top-emotions, resp.) is not only sufficient for a successful identification of the expression, but moreover, adding more emotional cues (namely, the other face half), does not cause a significant increase in performance level. Although it must be noted that a trend in that direction is visible in both groups. These findings could suggest that both groups of children did not integrate emotional information across both face parts, depending mostly on a more local analysis of the salient regions. Since our participant group consisted of young children—all the participants were younger than 10 years—it could indeed be that they tend to use a more feature-based recognition strategy in daily life too. However, it should be noted that the age at which children shift from using a primarily feature-based representation for faces to more holistic encoding processes is not uncontroversial [48, 49]. Although there are inconsistencies, some studies suggest that children with ASD use a more feature-based face processing strategy (for a recent overview, see [20]), which is in line with the neurocognitive theories attributing an atypical, more locally biased perceptual style to individuals with ASD (for two different views on this topic, see [50, 51]). Further research is necessary to explore the role of holistic processing in the emotion recognition in individuals with ASD and the developmental trajectory of this evolution.

This study did not provide evidence for the use of an atypical emotion recognition strategy in children with ASD: children with ASD were not found to rely more on information in the lower part of the face in comparison to TD children. These findings contrast with those of previous work which argued for the higher reliance on the mouth region in children with ASD ([16, 27, 35, 36], and also see, e.g., [37]). Apparent inconsistencies could be explained by several factors. Stimulus-specific aspects probably play an important role. Previous studies attributed a crucial influence to stimulus complexity, and most importantly, the embedding of the facial stimulus in a social context [3, 18]. It was sometimes suggested that the increased fixation on mouths can be linked to speech perception: children with ASD, and especially the group of children without language disorder, focused on the mouth region as a compensatory mechanism for their difficulties in processing eye information [52]. The use of dynamic and auditory material is consistent with the proposal that more naturalistic and ecologically valid paradigms should be developed in ASD research [27]. In addition, most previous work did not make a distinction between viewing patterns for different expressions, hereby disregarding that the significance of information in the eyes or mouth region could be strongly emotion dependent, as suggested by the difference between top- and bottom-emotions. In addition, compensatory mechanisms could also cause inconsistencies. Although one of the advantages of using hybrid stimuli is the fact that you get an insight into the implicit processing strategies, more cognition- or language-mediated compensatory mechanisms cannot be fully excluded. The unlimited presentation time (which maybe gave the children the time to use compensation strategies), the fact that the experimental group consisted of children without intellectual disability (deficits are more often found in younger children and in children with intellectual disability), the multiple choice response format, the types of stimuli, and the use of basic emotions, could make children to use these compensation strategies. One child, for example, commented while viewing an angry expression that “the eyebrows are turned downwards so this must be angry.” Please note that it is nearly impossible to exclude the possibility that the ASD sample used a more time-consuming, less efficient processing strategy. One could argue that designs with a limited presentation time partially exclude this. However, a confusion is created in these instances: it becomes very hard to distinguish between task difficulty and the use of time-consuming compensatory mechanisms. Whenever participants fail at a task using shorter presentation times, it becomes unclear whether this is due to the increased difficulty as such or because of an atypical default processing manner (i.e., a compensation strategy).

Employing an emotion labeling task with static emotional expressions, we found no evidence for an emotion recognition impairment in six-to-eight-year-olds with ASD. Furthermore, using hybrid stimuli, we demonstrated that the face region providing the most salient information is strongly emotion dependent. We hereby extended research findings on top- and bottom-emotions to children with and without a developmental disorder. This factor should be taken into account in future research, and the distinction between top- and bottom-emotions and the impact hereof on scanning patterns should be evaluated. In addition, we showed that coarse attention patterns can be evaluated with this innovative stimulus manipulation technique, which does not require eye-movement registration. Lastly, no evidence for a stronger mouth reliance in ASD was found in our task. Future research needs to investigate whether the same holds for more complex emotions and for emotions where information of both the upper and lower parts is required to achieve good recognition.

## Figures and Tables

**Table 1:** Demographic characteristics of both participant groups: means (*M*) and standard deviations (*SD*) for age (expressed in months), full-scale IQ, and ADI-R scores. Group differences in age or IQ were evaluated with independent samples *t*-tests (homoscedasticity assumption checked with *F*-test).

	ASD group (*n* = 22)		Comparison group (*n* = 22)		Group comparison	
	*M* (*SD*)	Range	*M* (*SD*)	Range	2-sided *t*-test	*P* value
Age	92.14 (10.54)	75–106	95.36 (8.16)	79–107	1.11	.2483
Full-scale IQ	94.36 (11.93)	78–119	98.50 (7.78)	84–108	1.39	.1816
ADI-R scores	44.50 (12.59)	15–65	N/A	N/A		N/A
Communication	15.68 (5.27)	3–24				
Social	20.45 (5.63)	8–28				
Repetitive behaviors	5.41 (2.65)	1–9				

**Table 2:** Accurate labeling of the expressions for both participant groups: mean accuracy and standard deviation (between brackets).

	ASD	TD
Anger	.71 (.22)	.67 (.26)
Fear	.76 (.21)	.75 (.21)
Happiness	.92 (.11)	.98 (.04)
Neutral	.60 (.31)	.76 (.13)
Sadness	.52 (.23)	.56 (.26)

**Table 3:** Accurate labeling of the hybrid expressions for both participant groups: mean accuracy and standard deviation (between brackets).

Emotion	Hybrid type	ASD	TD
Anger	Neutral eyes	.18 (.18)	.26 (.29)
	Neutral mouth	.62 (.21)	.74 (.13)
	Original	.71 (.22)	.67 (.26)

Fear	Neutral eyes	.35 (.16)	.50 (.16)
	Neutral mouth	.63 (.32)	.66 (.26)
	Original	.76 (.21)	.75 (.21)

Happiness	Neutral eyes	.86 (.16)	.80 (.31)
	Neutral mouth	.28 (.21)	.32 (.35)
	Original	.92 (.11)	.98 (.04)

Sadness	Neutral eyes	.25 (.19)	.26 (.15)
	Neutral mouth	.43 (.17)	.45 (.22)
	Original	.52 (.23)	.56 (.26)
